# Follow the money: a global analysis of funding dynamics for global health security

**DOI:** 10.1093/haschl/qxae083

**Published:** 2024-06-06

**Authors:** Hailey Robertson, Ellie Graeden, Justin Kerr, Michael Van Maele, Rebecca Katz

**Affiliations:** Center for Global Health Science and Security, Georgetown University, Washington, DC 20007, United States; Center for Global Health Science and Security, Georgetown University, Washington, DC 20007, United States; Center for Global Health Science and Security, Georgetown University, Washington, DC 20007, United States; Talus Analytics LLC, Boulder, CO 80301, United States; Center for Global Health Science and Security, Georgetown University, Washington, DC 20007, United States

**Keywords:** health financing, global health security, pandemic preparedness and response

## Abstract

Global financing for health security was dramatically impacted by COVID-19. Here, we provide an empirical analysis of how that funding changed. Using data from Global Health Security (GHS) Tracking (tracking.ghscosting.org), we analyzed disbursements of direct financial assistance for GHS from 2016 to 2022 to compare pre-pandemic funding (2016-2019) to post-pandemic funding (2020-2022) for preparedness and response during each of the seven World Health Organization-declared public health emergencies of international concern (PHEICs) from 2009 to 2022. Over $165B was disbursed for capacity-building and preparedness activities between January 2016 and December 2022, and over $76B was provided for PHEIC response. Preparedness funding remained evenly distributed since 2016 across regions, with the African region receiving about 70% of total preparedness funding. Indeed, how capacity-building and preparedness funding is distributed has changed remarkably little since 2016, despite unprecedented changes to the funding environment—including markedly increased spending—in response to COVID-19. This suggests we now have a unique opportunity to restructure how funds are tracked for accountability and assessing return on investment moving forward.

## Introduction

Significant efforts are underway to strengthen the global health security (GHS) architecture in the wake of the COVID-19 pandemic based on principles of equity, transparency, and accountability. As national and regional entities build out pandemic preparedness and response (PPR) capacities, existing global health funds, like the Global Fund to Fight AIDS, Tuberculosis, and Malaria, are redefining their scope to include pandemic preparedness efforts.^1-4^ To support these regional and national capacity-building efforts, the World Bank established the Pandemic Fund in 2022, which disbursed $337 million in support to single- and multi-country projects in the first round of proposals.^5,6^ The demand, however, is much greater, as signaled by the more applications from 133 countries requesting over $2.5 billion in support.^7^ Building and sustaining the GHS landscape requires new levels of funding from the global to the local level, as well as new approaches to determining what is financed, by whom, and when.^8^

To assess the relative scope of these investments and support analysis of the impact of these funds, the global community must better understand where financing was directed leading up to the pandemic, and how the global response efforts changed how these funds were invested and for what. Indeed, with changes underway to fund PPR for the next decade, an empirical analysis of these prior funding efforts is needed to guide sustainable investment for future outbreaks. Here, we present an analysis of funding in GHS and PPR between 2016 and 2022 to elucidate how the funding landscape has evolved since the end of Ebola in West Africa to the COVID-19 pandemic.

We observe a large increase in response-specific funding allocated toward COVID-19, as well as changes to the pathways through which financing occurs. However, we find that the distribution of capacity-building and preparedness funding—in terms of both who and what was funded, has experienced little change despite the rapidly evolving GHS landscape.

## Data and methods

Created in 2016, GHS Tracking (tracking.ghscosting.org) is a data platform providing information about the flow of international funding for health security. Building on existing funder-specific,^9^ disease-specific,^10^ and multi-sectoral^11,12^ financial trackers that capture health-related funding, the GHS Tracking tool captures $361 billion in disbursed funding through the end of 2022, including 1.6 million line-listing financial transactions over 130 000 projects across 1093 unique stakeholders.

To summarize previously published technical details about the tool's development,^13,14^ we aligned the data available on GHS Tracking to the Joint External Evaluation (JEE) 1.0. core capacity technical areas for preparedness funding. By mapping funding back to the JEE, we align investments to the national capacities that are assessed as part of the International Health Regulations (IHR), the governing framework for health security. This analysis did not include committed funds for which disbursements could not be verified or in-kind contributions, for which monetary value is not well-defined, nor was domestic health spending, as it is not routinely available.^15^ For preparedness funding, we analyzed disbursement data with transaction dates between January 2016 and December 2022, corresponding to the World Health Organization (WHO) adoption of the JEE 1.0. tool in January 2016.^16^

Funding specific to emergency response efforts was analyzed independently for each of the seven public health emergencies of international concern (PHEICs) declared by the WHO: 2009-2010 H1N1 influenza, 2014-2016 Ebola in West Africa, 2015-2016 Zika virus, 2014-present poliovirus, 2018-2020 Ebola in the Democratic Republic of Congo, 2019-2023 COVID-19 pandemic, and 2022-2023 Mpox. These events were chosen because they met the WHO's criteria for a global public health emergency that warranted a coordinated global response.

The total value disbursed by each funder for each recipient in the GHS Tracking database was computed using a structured query language query that aggregated transactions matching the filter conditions described above with separate queries for preparedness funds and response-specific funds. The relative percentage of funds disbursed was grouped by JEE thematic area of prevent, detect, and respond; core capacity; and WHO region for preparedness funds and PHEIC and WHO region for response-specific funds. The code and data to produce all figures can be found in a public GitHub repository (https://github.com/cghss/ghs-tracking-flows). All funding is reported in nominal US dollars.

## Results

**Figure 1.**
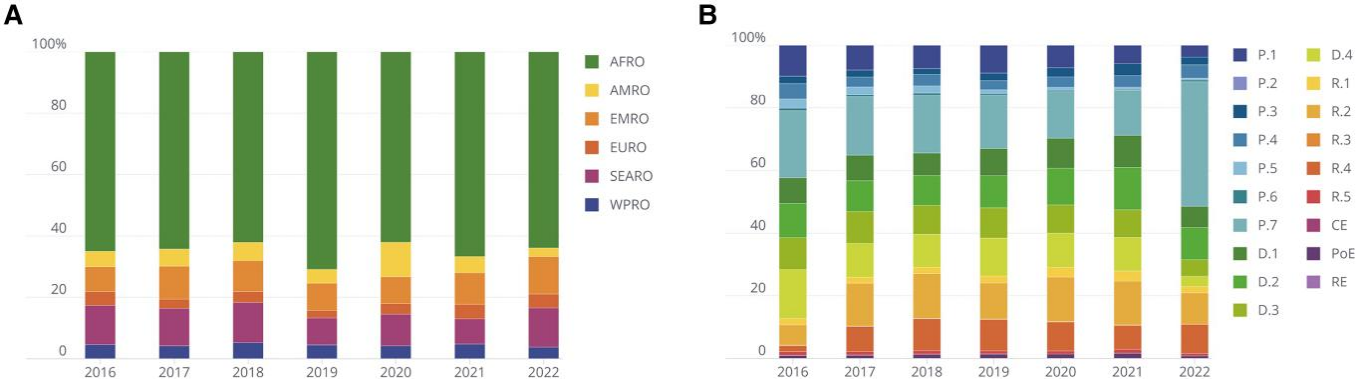
Preparedness funding distributed for global health security between January 2016 and December 2022. (A) Relative proportion of disbursed preparedness funding distributed to each World Health Organization (WHO) region including the African Region (AFRO), the Region of the Americas (AMRO), the Eastern Mediterranean Region (EMRO), the European Region (EURO), the Southeast Asian Region (SEARO), and the Western Pacific Region (WPRO). (B) Relative proportion of total preparedness funding distributed to each of the 19 International Health Regulations (IHR) Joint External Evaluation (JEE) 1.0. core capacity technical areas, grouped by thematic area - “Prevent” capacities (P.1-P.7), “Detect” capacities (D.1-D.4), “Respond” capacities (R.1-R.5), and “IHR-Related Hazards” (Ce, PoE, RE). The full name of each capacity can be found in the following notes. If a single transaction supported two or more capacities, the full transaction amount was counted for all capacities in both the numerator and denominator. Source: Underlying data available at tracking.ghscosting.org and code at https://github.com/cghss/ghs-tracking-flows. Notes: P.1, National Legislation, Policy and Financing; P.2, IHR Coordination, Communication, and Advocacy; P.3, Antimicrobial Resistance (AMR); P.4, Zoonotic Disease; P.5, Food Safety; P.6, Biosafety and Biosecurity; P.7, Immunization; D.1, National Laboratory System; D.2, Real-Time Surveillance; D.3, Reporting; D.4, Workforce Development; R.1, Preparedness; R.2, Emergency Response Operations; R.3, Linking Public Health and Security Authorities; R.4, Medical Countermeasures and Personnel Deployment; R.5, Risk Communication; PoE, points of entry; Ce, chemical events; RE, radiation emergencies.

We find that over $165B in international financing was disbursed for capacity-building and preparedness activities between January 2016 and December 2022, with over 51% of these funds being specifically aligned with the JEE. Funding across recipients for capacity-building and preparedness was consistently disbursed over time, with approximately 70% of total distributed funds going toward the African region, followed by around 10% of funding distributed to the Southeast Asian and Eastern Mediterranean regions (Figure 1A). Funding for the “Prevent”, “Detect”, and “Respond” categories of the JEE is also relatively evenly distributed between categories and across time (Figure S1). Investment in each of the JEE core capacities has been relatively stable since 2016 with the notable exclusion of P.7.—Immunization (Figure 1B), which increased from 15% of total funding in 2021 to 40% in 2022, corresponding to global COVID-19 vaccination efforts. P.7.—Immunization was also the top funded capacity overall, followed by R.2—Emergency Response Operations, D.2—Real-Time Surveillance, and D.4—Workforce Development. Funding remained largely consistent for these other top capacities from 2016 to 2022.

Similarly, we find little change in how funders distributed preparedness and response funds prior to and during COVID-19 with the exception of funding from international organizations to countries during the pandemic, which increased from 32% to 46% (Figure S2).

As described in previous work,^13^ funding for GHS capacity-building, as captured by the JEE, was largely driven pre-pandemic by monies from a few large country donors and international organizations. Indeed, almost 90% of all funding from 2016 to 2019 came from just the 10 top donors (see Table S1). However, as the COVID-19 pandemic unfolded, funding for response shifted this dynamic with significantly more funding spent by national governments to meet the need for public and medical health within their borders.^17,18^

**Figure 2.**
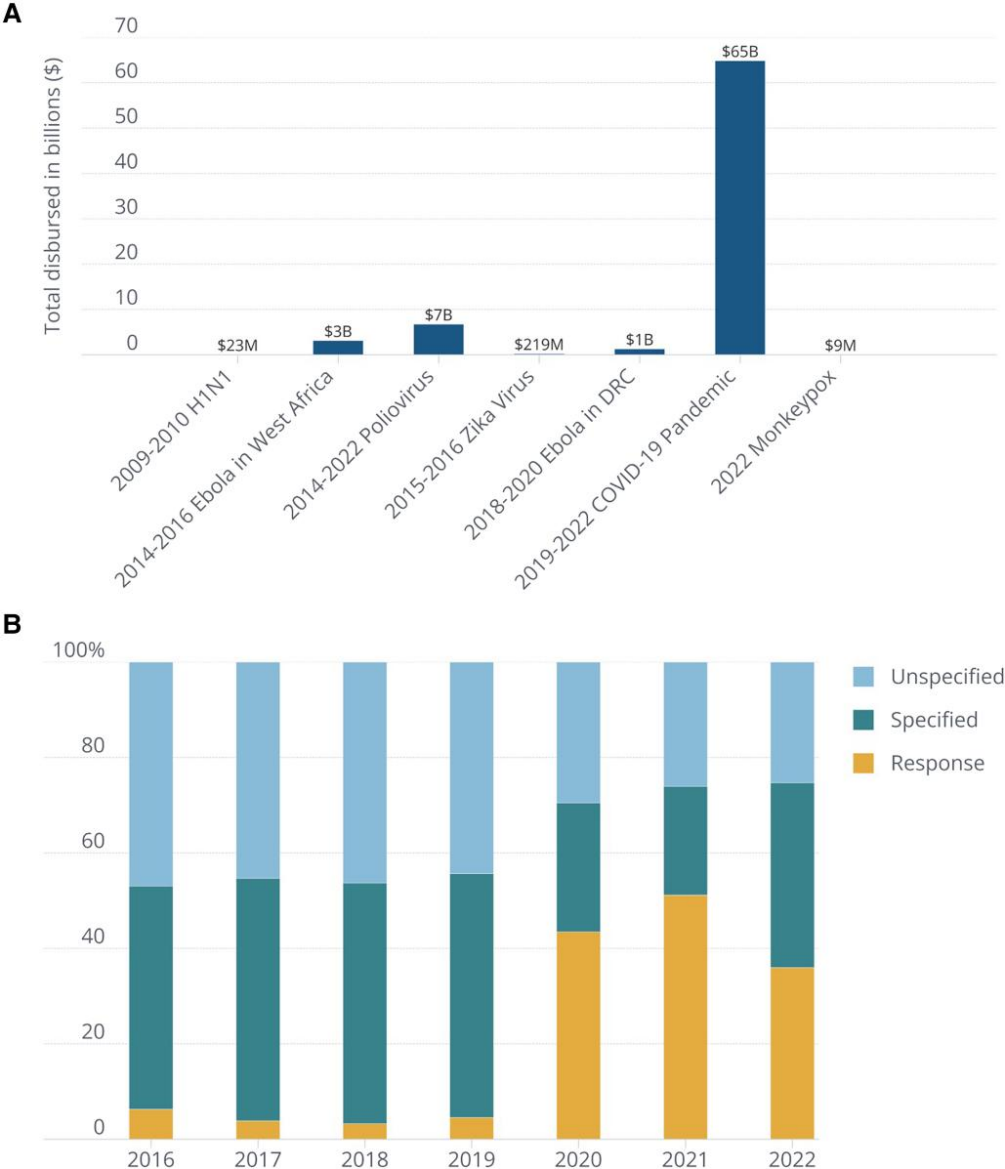
Total public health emergency of international concern (PHEIC) response international funding captured over time. (A) Total international response funding distributed in billions for each of the seven PHEICs declared by the World Health Organization, from 2009-2010 H1N1 influenza to 2022 Monkeypox (Mpox). (B) Relative proportion of global health security (GHS) funding allocated between January 2016 and January 2022 to specified activities (preparedness funding tagged with a Joint External Evaluation (JEE) core capacity technical area), unspecified activities (preparedness funding not tagged with a JEE core capacity technical area but supportive of, or cross-cutting with GHS objectives), and PHEIC response activities. Source: Underlying data available at tracking.ghscosting.org and code at https://github.com/cghss/ghs-tracking-flows.

Funding for PHEIC response has been substantial: Over $76B has been reported and disbursed as international response funding since 2009 (Figure 2A). Funding for PHEICs has ranged from $23 M reported for H1N1 in 2009-2010 to over $65B reported in response to COVID-19 from 2019 to 2022 (Figure 2A). Only a small percentage of total spending was allocated to specific response activities prior to COVID-19 (Figure 2A and B). Between 2016 and 2020, nearly 50% of funding from that period was instead allocated to efforts not specified in the JEE, including (1) HIV/AIDS, TB, and malaria-specific initiatives; (2) health sector reform programs; (3) vector control efforts; (4) women's health services; (5) livestock and veterinary programs; and (6) basic nutritional support. Outside of these areas, reported funds frequently provide so little information that the purpose of the project cannot be determined by researchers. Even following a significant increase in PHEIC-specific funding after 2020, these “unspecified” funds represent 28% of annual GHS spending on average. Funding efforts shifted significantly toward COVID-19 response starting in 2020, with 36% of total funding in 2020 and 46% in 2021 allocated to the PHEIC response (Figure 2A and B).

## Discussion

Although the global community has experienced multiple public health emergencies, governance initiatives, and a generation-defining pandemic since 2016, our results show that the global landscape for financing health security has changed remarkably little. There are, however, important new developments that may change the way GHS is funded going forward.

Firstly, almost all nations increased domestic spending on health security as part of the pandemic response. This appeared to be, in good part, driven by a new and immediately credible threat to the health of citizens at a scale not seen for much of the world in the decades prior. Therefore, to reduce the spread of COVID-19 and its downstream impacts to both health and healthcare systems in each country, governments invested heavily in domains that had previously been reserved for more general health care. This funding, because it was focused on pandemic response, is classified as GHS funding, but also reflected a greater focus on health care than had been previously captured as such. It will be critical to closely watch how much of that domestic spending is maintained and in what forms.

Secondly, the Pandemic Fund, the Global Fund, Gavi, the Vaccine Alliance, the Coalition for Epidemic Preparedness Innovations, and the new funding requests from the WHO may increase the overall size of financing in health security. As these entities increase in size and scope—including through the expansion of how the newer iterations of the JEE define health security capacity-building, we may see a decrease in bilateral funding. The evolving landscape of health security financing, particularly in light of how COVID-19 response efforts evolved, illustrates the importance of coordinating between horizontal and vertical funding programs. All financing, whether it is for a disease-specific program or system strengthening for all health efforts, should be coordinated where possible and endeavor to integrate into a single health system. This is an opportunity for better integration between the health sector and health security.

Thirdly, the JEE does not currently have explicit categories for important cross-cutting capacities and focus areas (eg, HIV/AIDS, malaria, TB, maternal/child health, nutritional support, health policy reform, etc.), even though many of the core capacities outlined in the latest iterations of the JEE can benefit these programs.^19^ Similarly, funding for TB programs can directly and indirectly benefit health security when properly integrated.^20^ This integration is an area for the development of standardized PPR categories that emphasize both a One Health and universal health coverage approach.

Critically, we must identify ways to capture how response funding can sustain and build upon local capacity-building efforts such that the capability is not lost when the emergency expires, as has historically been the case with some short-term projects in global health.^21^ Some response funding is inevitably for consumable items that are only used for a short period of time, like the procurement of a medical countermeasure. Yet, the funding may also be used to support delivery systems of medical countermeasures, which may be sustained and used in the future, thus contributing to national capacity-building. We see this most apparently with investment in JEE P.7—Immunization, where funding for capacity-building expanded in tandem with COVID-19 vaccination response efforts (see Figure 1B). Similarly, response funding used to rapidly train a clinical workforce in critical infection, prevention, and control for an outbreak is knowledge that is maintained by that local workforce and can be applied to future outbreaks.^22^ However, our findings do not demonstrate increased investment in workforce development or personnel deployment during COVID-19, and these areas have been regularly underfunded.^23,24^ Future research should analyze which capacities were sustained as a result of COVID-19 and determine the role that international funding vs domestic funding played in this capacity-building. Doing so will greatly benefit governments and donors. However, there is currently no effective way to capture or validate how much response funding contributes to capacity-building in part due to a lack of detailed, standardized public reporting about funding and implementation—especially domestic allocations.

Finally, fully understanding and tracking funds in health security necessitates improved, standardized measurements of domestic spending. Implementing PPR funding categories into national health accounts will help to address this challenge.

Few other global efforts to track funding for the COVID-19 pandemic with a focus on grants have been completed from which to benchmark the estimates presented here. The United Nations Office for the Coordinator of Humanitarian Affairs (UNOCHA)'s Financial Tracking Services COVID Humanitarian Response Plan captures just under $4B committed for COVID-19 response through United Nations (UN) pledges; our data capture a broader subset of donor funding.^25^ The Global Burden of Disease study by the Institute for Health Metrics and Evaluation reported that $38B was provided for health-related COVID-19 response between January 2020 and December 2021, which is <65% of the funds we identified using self-reporting from countries and other donors in the same period, totaling $60B.^26^ By contrast, the Centre for Disaster Protection estimated $80B in distributed aid from 2020 alone though this includes lending and bilateral debt repayment suspension data, not included in this analysis.^27^

## Conclusion

Funding and financing for GHS in the decade leading up to the COVID-19 pandemic arguably had a significant impact on our ability to respond effectively to a global threat.^21^ There is a great need for continued financing in health security to build and sustain core capacities for preparedness and response, as well as a need for response funding, particularly as all indicators point to increased probabilities of more public health emergencies around the world.

This study is the first to assess how this funding changed over time, including a comparison to a pre-pandemic world and including analysis not just of response funding, but of funding and financing of preparedness efforts that underpin effective response. This study draws from a unique dataset that provides the only comprehensive assessment of funding for GHS over the last decade and assesses how this funding aligns with the JEE across preparedness domains.

Findings from this analysis suggest that funders have not yet shifted their investment strategies to account for lessons learned from the pandemic—such as the need to invest in sustainable capacity-building via workforce development and health system strengthening—even as the global health landscape has shifted dramatically. The findings also indicate the scale to which a vast number of stakeholders mobilized in response to COVID-19, especially as more was being spent by countries internally. However, we lack the data and public reporting to assess whether these funds have or will translate into sustained future capacity and pandemic preparedness, and to what extent.

As the world emerges from the pandemic, we need to find new and more effective ways of identifying and tracking investments in global health—including national spending account activity and One Health investments. Particularly as new funding platforms and mechanisms from the Pandemic Fund and beyond emerge, there are critical concerns about how funds will be directed and whether financing models can be developed that account for the lessons of the past decade.^21,23,26,28^ In light of these developments, we need to ensure we understand how financing flows, what are the best mechanisms for success, and how to ensure funding from international, bilateral, and domestic sources is equitable, sustainable, and seamlessly integrated into health systems.

## Supplementary Material

qxae083_Supplementary_Data
