# Supplementary material for: Follow the money: a global analysis of funding dynamics for global health security
Source: Health Aff Sch. 2024 Jun 6;2(6):qxae083. doi: 10.1093/haschl/qxae083 (PMC11196183; doi:10.1093/haschl/qxae083)
Supplement: qxae083_Supplementary_Data [file qxae083_supplementary_data.zip › Appendix 1.docx]

# **Appendix**

## **Supplementary Table 1. Top 25 funders by total funding disbursed for capacity from 2016-2019**

*Note there are over 400 funders captured in the GHS Tracking database

|  | **Funder name** | **Total USD disbursed by funder** | **Share (%) of total funding disbursed** |
| --- | --- | --- | --- |
| 1 | United States of America | $30,189,807,002.43 | 32.104 |
| 2 | The Global Fund to Fight AIDS, Tuberculosis and Malaria | $14,947,768,876.28 | 15.896 |
| 3 | Bill & Melinda Gates Foundation | $9,137,784,245.69 | 9.717 |
| 4 | United Nations (UN) | $5,802,494,891.25 | 6.170 |
| 5 | World Bank | $5,598,649,460.18 | 5.954 |
| 6 | Global Alliance for Vaccines and Immunisation (GAVI) | $5,288,030,121.78 | 5.623 |
| 7 | United Kingdom | $5,113,613,546.95 | 5.438 |
| 8 | European Commission - International Partnerships | $2,678,754,564.20 | 2.849 |
| 9 | Canada | $2,550,681,904.76 | 2.712 |
| 10 | Netherlands | $1,982,797,651.11 | 2.109 |
| 11 | Germany | $1,902,215,460.49 | 2.023 |
| 12 | Inter-American Development Bank | $984,168,315.00 | 1.047 |
| 13 | Australia | $978,547,493.83 | 1.041 |
| 14 | Norway | $830,035,268.40 | 0.883 |
| 15 | World Health Organization (WHO) | $787,815,893.80 | 0.838 |
| 16 | Asian Development Bank | $679,534,309.43 | 0.723 |
| 17 | Sweden | $618,002,066.97 | 0.657 |
| 18 | Italy | $287,854,112.40 | 0.306 |
| 19 | Denmark | $275,121,471.32 | 0.293 |
| 20 | France | $240,165,025.14 | 0.255 |
| 21 | Sightsavers | $200,358,758.48 | 0.213 |
| 22 | King Salman Humanitarian Aid and Relief Centre | $196,583,374.00 | 0.209 |
| 23 | MSI Reproductive Choices | $192,585,714.35 | 0.205 |
| 24 | Finland | $159,986,366.50 | 0.170 |
| 25 | Malaria Consortium | $144,149,393.57 | 0.153 |

**[INSERT Supplementary Figure 1 HERE]**

**Supplementary Figure 1.**

**Caption:** Relative proportion of total preparedness funding distributed to each of the JEE thematic categories (“Prevent”, “Detect”, and “Respond” ) by year from 2016-2022.

**SOURCE**: Underlying data available at tracking.ghscosting.org and code at https://github.com/cghss/ghs-tracking-flows.

**NOTES**: When a single transaction supported two or more capacities, the full transaction amount was counted for all capacities in both the numerator and denominator.

**[INSERT Supplementary Figure 2 HERE]**

### **Supplementary Figure 2.**

### **Caption:** Flows of total disbursed funding (response and preparedness) from funders to recipients.“Country” describes foreign assistance to/from a national government or its agencies; this does not include any domestic spending or national health account line items. “International” refers to funding to/from international organizations, including both international governmental organizations (e.g., the United Nations) and non-governmental organizations (e.g., Doctors Without Borders). “Other” includes funding to/from private sector stakeholders, public-private partnerships, and academic organizations. “Philanthropy” refers to funding to/from charitable foundations and trusts. (A) Funding pathways for flows disbursed by funders (left) to recipients (right) from January 2016 to December 2019, before COVID-19. (B) Funding pathways for flows disbursed by funders (left) to recipients (right) from January 2020 to December 2022, during COVID-19.

SOURCE: Underlying data available at tracking.ghscosting.org and code at https://github.com/cghss/ghs-tracking-flows.

###

### 
